# Utilization of ultrasonic aspirator for combined aortic and mitral valve decalcification: a case study

**DOI:** 10.1186/s13019-024-03324-3

**Published:** 2025-01-23

**Authors:** Mohammad Alomari, Breah Paciotti, Pankaj Garg, Sibat Noor, Nafiye Busra Celik, Basar Sareyyupoglu

**Affiliations:** Department of Cardiothoracic Surgery, Mayo Clinic, Jacksonville, FL USA

**Keywords:** Mitral annular calcification (MAC), Mitral valve replacement (MVR), Aortic calcification, Decalcification, Sonopet

## Abstract

Mitral and aortic annular calcification is an age-related degenerative process that can result in severe mitral and/or aortic stenosis and/or regurgitation. Annular calcification not only increases the surgical complexity but also increases the risk of complications. In this case report, we present the innovative use of the Sonopet ultrasonic surgical aspirator for aortic and mitral annular decalcification in a patient with hypertrophic obstructive cardiomyopathy, mild aortic stenosis and moderate mitral regurgitation in the presence of mitral annular calcification (MAC) and aorto-mitral curtain calcification.

**Supplementary Information:**

The online version contains supplementary material available at 10.1186/s13019-024-03324-3.

## Introduction

The ultrasonic aspirator, a device capable of fragmenting tissue via ultrasonic waves without damaging adjacent structures, was originally developed for neurosurgical procedures. this device by high-frequency vibration breaks up the brittle calcium without damaging the underlying soft tissue as confirmed by the histopathological examination of the excised valves [1, 2]. Simultaneous feeding of the aerosolized water prevents the damage to the adjacent tissues due to the heat generated by the device. Although, in recent years, ultrasonic devices have made some strides in the successful decalcification of the mitral and aortic annulus in patients with mitral annular calcification (MAC) and Aortic stenosis (AS) [3, 4], their application in cardiac surgery still remains underutilized. We herein report the use of Sonopet ultrasonic device for the simultaneous decalcification of the MAC and aorto-mitral curtain in a patient undergoing surgical septal myectomy, aortic valve repair and mitral valve replacement (MVR).

## Case

A 75-year-old lady with history of hypertension, hypertrophic obstructive cardiomyopathy, atrial fibrillation, pulmonary embolism, and myocardial infarction, S/P percutaneous coronary intervention (PCI), presented with progressive fatigue and exertional dyspnea for one year. Transthoracic echocardiogram revealed restricted left coronary cusp with mild AS with mild AR, and moderate MR with mild mitral stenosis in the setting of severe MAC. Left ventricular (LV) end-diastolic diameter was 60 mm, and LV ejection fraction (LVEF) was 56%. Coronaries were normal on coronary angiography. Non-contrast computed tomography of the chest also confirmed severe MAC.

## Operative technique

**Fig. 1:**
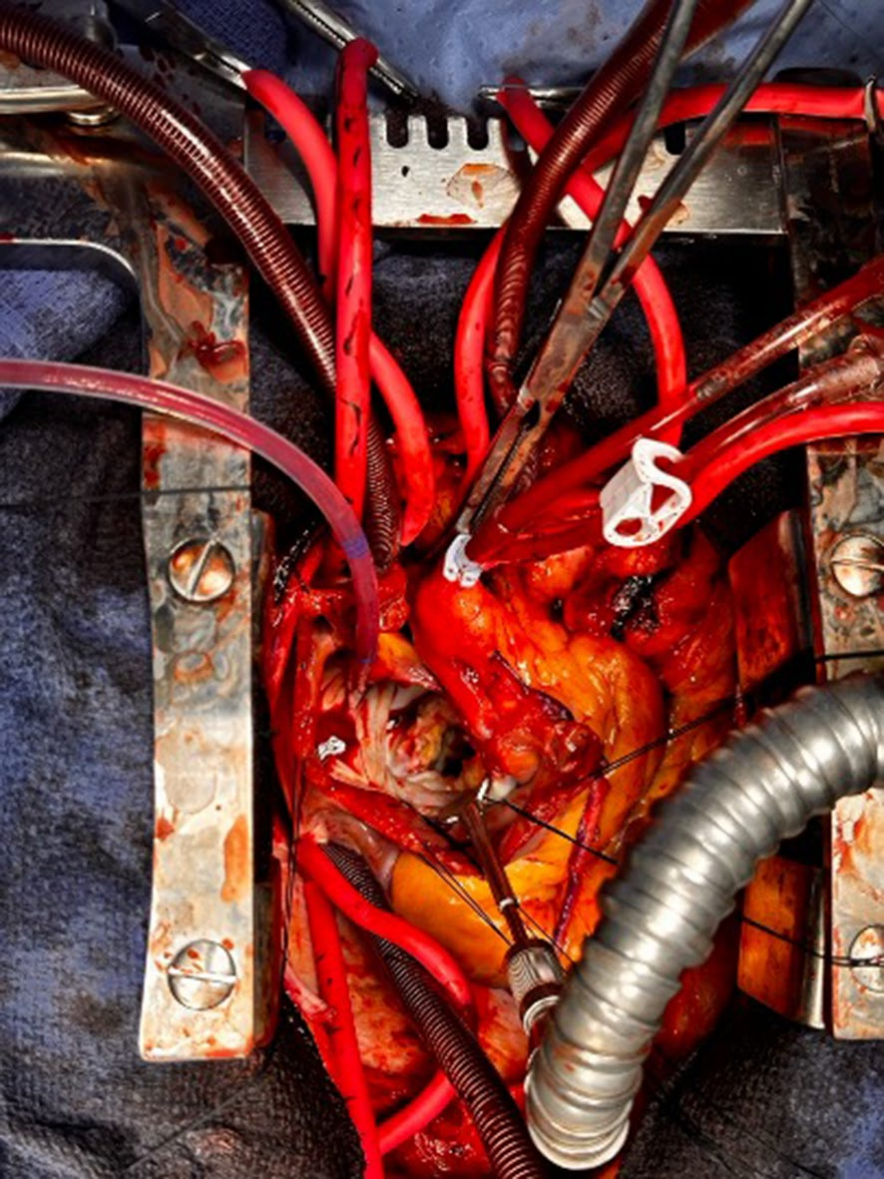
Intraoperative photograph showing severe mitral annular calcification along the posterior annulus

Surgery was performed on mild hypothermic cardioplegic arrest. Aortotomy was performed to access the aortic valve. Left aortic cusp was frozen due to calcified fibrous trigone. Initially, we performed the septal myectomy using a PlasmaBlade [5]. Subsequently, calcium from the fibrous trigone under the left aortic cusp was debrided using Sonopet ultrasonic aspirator (Stryker(^®^), Kalamazoo, MI, USA). Decalcification significantly improved the mobility and coaptation of the aortic cusps. Subsequently, aortotomy was closed in two layers and the mitral valve was approached through a trans-septal approach. Mitral valve was inspected but deemed unsuitable for repair; hence, the mitral valve leaflets were excised. There was extensive MAC along the posterior mitral annulus (Fig. 1). We debrided calcium from the mitral annulus using Sonopet ultrasonic aspirator with regular assessment of the mitral annulus (Video 1). Our aim was to achieve enough suppleness of the posterior mitral annulus to safely pass the needle for pledgeted sutures. Subsequently, 27 mm Epic bioprosthetic mitral valve (St. Jude Medical Inc, St Paul, MN, USA) was sutured. Subsequently, the inter-atrial septum and right atriotomy were repaired, and the aortic cross-clamp was removed. Patient was weaned off cardiopulmonary bypass on minimal inotropes. Transoesophageal echocardiography demonstrated well-functioning mitral bioprosthesis without paravalvular leak. Native aortic valve was also functioning well with minimal AR. Her postoperative course was uneventful, and patient was discharged on postoperative day 5. At last follow-up 1 year after surgery, patient remained asymptomatic with a well-functioning aortic valve and mitral prosthesis.

## Discussion

Mitral or aortic valve repair, whenever feasible, is preferred over replacement regardless of LV function and concurrent cardiac operations [2]. However, associated annular calcification makes the valve repair as well as replacement challenging. Aortic and mitral annular calcification, and aorto-mitral curtain calcification is an age-related degenerative process of the fibrous skeleton of the heart [6]. During MVR, MAC has been linked with the poor outcomes and increased mortality due to paravalvular leak, heart block, calcium embolization, stroke, left atrioventricular groove disruption due to overzealous mitral annular calcium debridement, and myocardial infarction due to the left circumflex coronary artery injury [1, 7].

Mechanical debridement of MAC results in improved mobility of mitral leaflets and annular pliability for the suture placement. Conventionally, mitral annular calcium is removed mechanically using pituitary rongeurs. However, in the presence of extensive annular calcification, mechanical debridement may result in significant loss of annular tissue, injury to adjacent myocardium, and the risk of annular disruption [8]. Similarly, mechanical debridement of the aortic valve has been described for calcific AS; however, recurrences of AS and the development of aortic regurgitation (AR) led to the abandonment of this practice [3].

The use of the Cavitron Ultrasonic Surgical Aspirator (CUSA) was first described by Hodgson et al. in 1979 [9]. all ultrasonic devices focus low-frequency ultrasound vibrations on the calcium specks, creating very small fragments that are aspirated while protecting surrounding soft tissue. Therefore, it helps in safe and effective debridement of MAC and aorto-mitral curtain calcification without disrupting the annular integrity. There are reports of using CUSA [6] and Sonopet Ultrasonic surgical aspirator for severe MAC during MVR for MS [4]. Brescia et al. [4]. conducted a retrospective comparative analysis focusing on patients operated for mitral valve disease and had MAC. The study observed no perioperative mortality or stroke in the Sonopet group (*N* = 15) compared to 10% (17/164) incidence of mortality (*P* = 0.37) and 7% (12/164) incidence of stroke (*P* = 0.60) in the non-Sonopet group. The difference however was not statistically significant. Among the 17 mortalities in the non-Sonopet group, AV groove dehiscence or rupture, multisystem organ failure, right heart and respiratory failure, massive stroke leading to uncal herniation, hemorrhage with ventricular fibrillation arrest, and aspiration with pneumonia were cited as the primary cause. Median length of hospital stays, mean mitral prosthetic gradient, and rate of reoperation (Sonopet group (7%) and non-Sonopet group (5%); *P* = 0.55) was not significantly different between the groups. Only one patient in the Sonopet group had mild MR, and no patients exhibited moderate or severe MR.

Ours is the first reported case of Sonopet use for both aortic fibrous trigone and posterior mitral annular calcium debridement. We believe that the use of Sonopet in cardiac surgery is significantly underutilized. In two other reports published from our institute, we successfully used the Sonopet ultrasonic surgical aspirator for pericardial cyst decalcification in a patient undergoing excision of calcified pericardial cyst [10] and difficult insertion of HeartMate 3 left ventricular assist device in a patient with severely calcified left ventricular apex [11]. The above-stated experience clearly demonstrates that Sonopet ultrasonic surgical aspirator can be safely used in cardiac surgery in various locations to decalcify while preserving the native cardiac tissue. Additional studies are warranted to further define role Sonopet for cardiac decalcification at various locations and its impact on the outcomes of cardiac surgery.

## Conclusion

Sonopet ultrasonic surgical aspirator is a safe and effective tool in debriding annular calcium and preserving adjoining cardiac tissue in patients with calcified aortic and/or mitral annular calcification.

## Electronic supplementary material

Below is the link to the electronic supplementary material.

Supplementary Material 1: Video 1. Intraoperative short clip, highlighting the posterior mitral annular decalcification using Sonopet ultrasonic machine.

## Data Availability

No datasets were generated or analysed during the current study.

## Declarations

### Permission

The patient signed a written informed consent regarding publication of this material inclining video and images.

## Abbreviations

AR: Aortic regurgitation
AS: Aortic stenosis
CUSA: Cavitron Ultrasonic Surgical Aspirator
MAC: Mitral annular calcification
MR: Mitral regurgitation
MVR: Mitral valve replacement
